# Pre-formulation and systematic evaluation of amino acid assisted permeability of insulin across *in vitro* buccal cell layers

**DOI:** 10.1038/srep32498

**Published:** 2016-09-01

**Authors:** Affiong Iyire, Maryam Alayedi, Afzal R. Mohammed

**Affiliations:** 1 Aston Pharmacy School, Aston University, Birmingham, B4 7ET, UK.

## Abstract

The aim of this work was to investigate alternative safe and effective permeation enhancers for buccal peptide delivery. Basic amino acids improved insulin solubility in water while 200 and 400 μg/mL lysine significantly increased insulin solubility in HBSS. Permeability data showed a significant improvement in insulin permeation especially for 10 μg/mL of lysine (p < 0.05) and 10 μg/mL histidine (p < 0.001), 100 μg/mL of glutamic acid (p < 0.05) and 200 μg/mL of glutamic acid and aspartic acid (p < 0.001) without affecting cell integrity; in contrast to sodium deoxycholate which enhanced insulin permeability but was toxic to the cells. It was hypothesized that both amino acids and insulin were ionised at buccal cavity pH and able to form stable ion pairs which penetrated the cells as one entity; while possibly triggering amino acid nutrient transporters on cell surfaces. Evidence of these transport mechanisms was seen with reduction of insulin transport at suboptimal temperatures as well as with basal-to-apical vectoral transport, and confocal imaging of transcellular insulin transport. These results obtained for insulin are the first indication of a possible amino acid mediated transport of insulin via formation of insulin-amino acid neutral complexes by the ion pairing mechanism.

The primary driving factor for investigating non-invasive routes for delivering biologicals is built on reducing/removing the need for single/multiple daily injections, which puts a major strain on patient compliance and desired therapeutic outcome^1^. On the other hand, mucosal delivery of biologicals has been reported to be less effective than parenteral delivery for various reasons including limited mucosal permeation, as well as absorption site metabolism^2^. However, high vascularisation and presence of fewer proteolytic enzymes in the buccal mucosa compared to the gastrointestinal tract (GIT) mucosa promotes buccal delivery as a potential site for protein and peptide delivery. Limitations of buccal delivery of proteins include: large molecular weight and hydrophilicity leading to low diffusivity; instability, fast metabolism, adsorption, aggregation and possible immunogenicity^2^. Thus factors affecting peptide drug absorption including structure (molecular weight/size, conformation, stereo-specificity, immunogenicity and electrostatic charge); physicochemical properties (solubility, hydrophilicity/partition coefficient, aggregation, self-aggregation and hydrogen bonding); buccal mucosal properties (structure and biochemistry); biological environment (enzyme sensitivity and intracellular metabolism) and available transport mechanisms have to be considered during buccal formulation development.

Strategies such as chemical modification by derivatisation, or prodrug approaches may enhance peptide stability and lipophilicity, and may prevent degradation by proteolytic enzymes at the mucosal surface^2^. Other strategies that result in the formation of non-covalent complexes between the protein and hydrophilic moieties have been reported to impart slight reversible unfolding to the peptide molecule, leading to enhanced protein flexibility and lipophilicity^1^,^3^. This increases passive transcellular diffusion and enhances protein permeability, and is the principle behind the use of ion pairs to enhance drug permeation. An ion pair is formed when a pair of oppositely charged (counter) ions are held together without the formation of a covalent bond; forming a neutral molecule with higher lipophilicity, that can partition into the cell membrane easier than the parent compound^4^. Most ion pairing agents such as inorganic surfactants and co-solvents require high volumes, resulting in high toxicity/allergenicity and have limited applications in pharmaceutical preparations^4^,^5^,^6^. Recently, the use of amino acids as ion pairs to enhance solubility/permeability of small molecules has been investigated as a biodegradable, low toxicity/irritant and high stability alternative^6^. Moreover, the availability of amino acid transport across cells may enhance facilitated transport of drug-amino acid complexes (after ion pair formation), leading to enhanced permeation^7^. Various studies have investigated the use of amino acids as ion pairs to improve solubility/permeability of small molecules^4^,^8^,^9^,^10^,^11^. However the use of amino acids as ion pairs to enhance solubility/permeability has not been extensively reported for proteins and macromolecules. Usually, with small molecules, molar ratios of the counter ion are used in excess^5^, but molar ratios cannot be employed for macromolecules because these would require minute quantities of amino acids which in turn would produce negligible improvements. Also, the large size, amphiphilic nature, and presence of multiple ionisable sites on proteins further complicate the expected results *in vitro*.

In order to establish the role of ion pairs in the formulation of biologicals, the proposed work is built on insulin as a model compound. Insulin has high solubility (depending on solution pH) and low permeability, mostly due to its large molecular weight, low lipophilicity and degradation in the presence of proteases^12^. The insulin monomer consists of six basic groups and six acidic groups which become ionised depending on solution pH^13^. The current work was based on two hypotheses. Firstly, that ionised insulin molecules could form ion pairs with opposite charges provided by basic and acidic amino acids to form an insulin-amino acid neutral complex with enhanced lipophilicity, which could then permeate the buccal membrane via transcellular passive diffusion. Secondly, that complexation of insulin with amino acids could possibly trigger alternate facilitated transport mechanism of insulin-amino acid complex across buccal membranes. Thus the aim of this work was to investigate the ion pairing effect of basic and acidic amino acids on the solubility, permeability, mechanism and the transport route of insulin across TR146 buccal cell layers.

## Results and Discussion

### Insulin solubility in water

There is a lack of consensus on solubility of insulin in water as different authors have either reported it as soluble/hydrophilic^15^ or insoluble/hydrophobic^16^. However, the solubility of insulin is determined by the pH of the solution, and its closeness to the isoelectric point of insulin^17^. Kramer *et al*.^18^ described protein solubility as a thermodynamic parameter defined as “the concentration of protein in a saturated solution that is in equilibrium with a solid phase, either crystalline or amorphous, under a given set of conditions”. This solubility depends primarily on intrinsic factors such as characteristics of its constituent amino acids, as well as extrinsic factors such as temperature, pH, ionic strength and solvent additives^18^. Our results show that insulin remained only slightly soluble in deionised water (<3 μg/mL) (pH 6.64). Similar results were obtained by Lens^17^ who measured insulin solubility at pH 6.0. Landreh^19^ also reported low insulin solubility at pH 5–6. The insulin monomer consists of six basic groups (two N-terminal amino groups, two histidine, one lysine and one arginine) and six acidic groups (two C-terminal carboxylates and four glutamates)^13^. Therefore at pH values around its isoelectric point of 5.35, charges on the monomer balance out providing a neutral molecule, rendering insulin practically insoluble in water^4^,^14^. At acidic pH, the acidic groups on insulin become protonated, and the resulting charge on the molecule is provided by the basic groups (NH_3_^+^), thus insulin has a positive charge in acidic media. However, in basic media, the acidic groups lose their protons and the charge on the monomer is provided by the acidic groups (COO^−^), giving insulin a negative charge in basic media. At the near neutral pH of deionised water (pH 6.6), the charges on the acidic and basic groups of insulin balance out to give a neutral molecule with no net charge, resulting in low solubility of the protein^4^,^14^.

### Insulin solubility in HBSS

Insulin solubility in HBSS was determined because it was expected that at pH 7.4 (more than 2 units above the isoelectric point of insulin) the protein would be soluble thereby overcoming precipitation^20^. HBSS also enabled the circumvention of the potential toxicity related to solubilising insulin in 0.01N HCl prior to permeation experiments as reported by other authors^21^,^22^. For HBSS solubility studies, a range of ionisable amino acids was utilised, their physicochemical properties are presented in Supplementary Table S1. As expected, insulin was highly soluble in HBSS (pH 7.4) as reported by Lens^17^. In the presence of basic amino acids (Supplementary Fig. S1), arginine and lysine were able to increase solution pH from 7.4 to 9.2 at concentrations above 100 μg/mL, while histidine caused no change in solution pH (pKa of the histidine side chain (6.04) causes it to act neutral or slightly basic at neutral pH and thus could not affect solution pH). However, only lysine was able to significantly enhance insulin solubility in HBSS from concentrations 200 μg/mL and above. This disparity in solubility enhancement for the basic amino acids is related to the isoelectric points of these amino acids and the basicity of their ionisable side chains. Elshaer *et al*.^7^ also reported higher solubility enhancement for indomethacin by lysine than arginine due to the self-association that exists between lysine and the drug. In the presence of the acidic amino acids (Supplementary Fig. S2), a reduction in solution pH was observed at amino acid concentrations above 200 μg/mL (due to the buffering capacity of HBSS); and a subsequent reduction in solubility was observed at these concentration (formation of a cloudy solution on visual examination) which were close to the isoelectric point of insulin^23^.

The effect of amino acids on insulin solubility was compared to that of sodium deoxycholate (Na deoxycholate), a hydrophobic bile salt that has been widely studied as a permeation enhancer. This is a surfactant that enhances permeability of biological membranes by dissolution of membrane lipids thereby interfering with membrane fluidity and integrity^24^,^25^,^26^,^27^. As a detergent, Na deoxycholate acts as an amphiphile and possesses both polar and non-polar properties and is able to enhance the emulsification of insoluble substances by reducing interfacial surface tension^28^. Na deoxycholate (pKa 6.58) had no effect on solution pH even at the highest concentrations. However, a significant reduction in insulin solubility was observed at 100 μg/mL Na deoxycholate. This was because the bile salt has been shown to confer a concentration - dependent hydrophobicity on proteins like insulin, thereby enhancing its lipophilicity, which would reduce aqueous solubility^29^.

Therefore, it can be concluded that the amino groups of the basic amino acids and the carboxyl groups of the acidic amino acids have the capacity for ionisation, and affect insulin solubility due to the ease of formation of ion pairs and soluble complexes in the presence of counter ions furnished by ionisable sites on the insulin moiety. This effect was dependent on the resultant system pH, pKa of the amino acids and the differences between these values and the isoelectric points of both insulin and the amino acids^14^.

### Insulin permeability across TR146 cell layers

Ion pairs can be formed in the presence of oppositely charged ions in solution. Polypeptides such as insulin with multiple potentially ionisable regions depending on the amino acid sequence and system pH, have the potential of forming ion pairs with amino acid side chains, to increase lipophilicity and thus may enhance permeability^30^. TR146 cells were grown on transwell inserts for 28–29 days before use for permeability experiments. Integrity of the stratified layers was monitored using TEER as shown in Supplementary Fig. S3. TEER results showed the formation of a stable stratified layer between days 28 to 29 which were used for experiments, as reported by other authors^21^.

The permeation of insulin across TR146 cell layers was tested in the absence/presence of increasing concentrations of the three basic (arginine, lysine and histidine), two acidic (aspartic acid and glutamic acid) amino acids and Na deoxycholate. Amino acids with ionisable side chains were chosen to facilitate ion-pairing with insulin via the counter ion mechanism; insulin is amphiphilic and can behave as an acid or a base depending on solution pH. Na deoxycholate was chosen as a model surfactant that has been well established in literature as a permeation enhancer, to determine if similar or better enhancement effects could be obtained from amino acids, without the toxicity issues presented by surfactants. The maximum amount of insulin that permeated the cells in the absence of amino acids (control) was 4.07 ± 0.82% after 4 hours.

#### Basic Amino Acids

**Figure 1:**
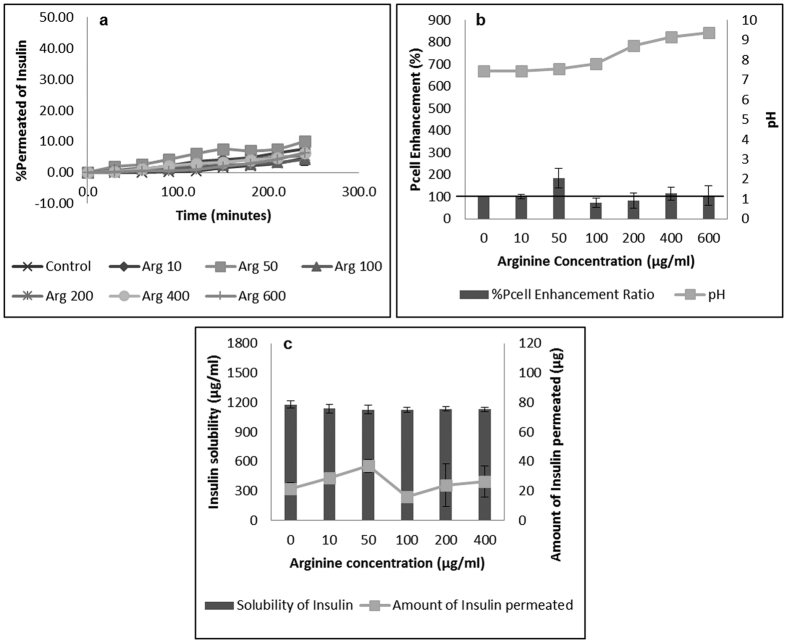
Permeation of human insulin across TR146 buccal cell layers in the presence of increasing arginine concentrations. (**a**) Percentage of insulin permeated in the presence of various concentrations of arginine. (**b**) Comparing the percent enhancement of insulin permeability through TR146 cell layers in the presence of various concentrations of arginine and solution pH. (**c**) Comparing the solubility at 25 °C and permeability of insulin through TR146 cells layers, of insulin in the presence of various concentrations of arginine.

**Figure 2:**
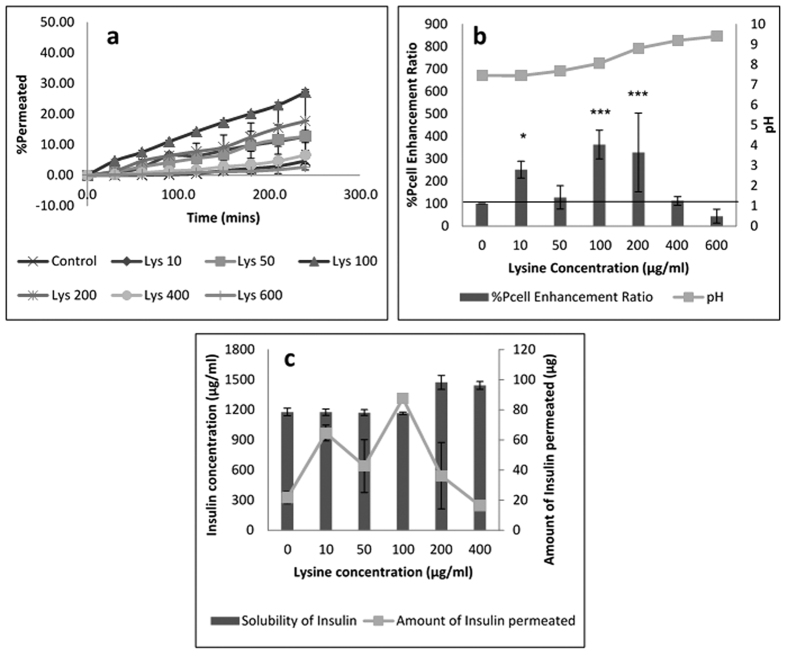
Permeation of human insulin across TR146 buccal cell layers in the presence of increasing lysine concentrations. (**a**) Percentage of insulin permeated in the presence of various concentrations of lysine. (**b**) Comparing the percent enhancement of insulin permeability through TR146 cell layers in the presence of various concentrations of lysine and solution pH. (**c**) Comparing the solubility at 25 °C and permeability of insulin through TR146 cells layers, of insulin in the presence of various concentrations of lysine.

**Figure 3:**
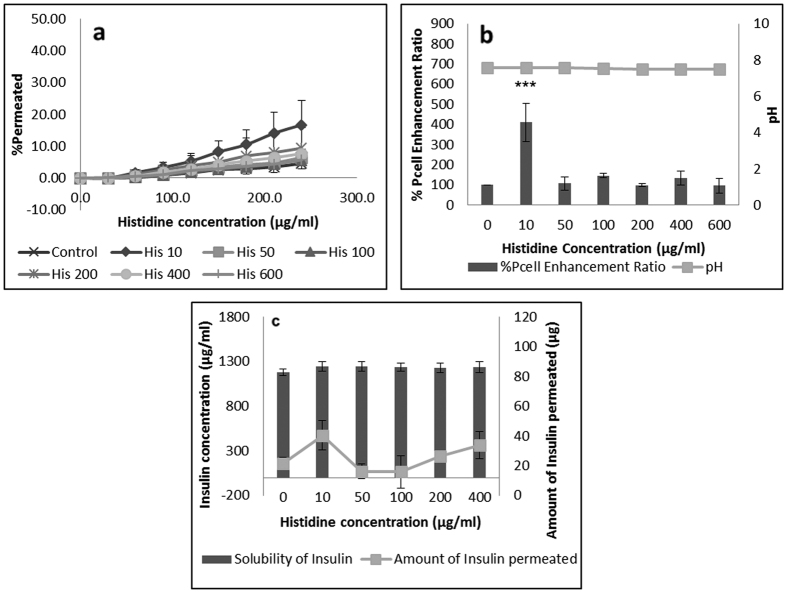
Permeation of human insulin across TR146 buccal cell layers in the presence of increasing histidine concentrations. (**a**) Percentage of insulin permeated in the presence of various concentrations of histidine. (**b**) Comparing the percent enhancement of insulin permeability through TR146 cell layers in the presence of various concentrations of histidine and solution pH. (**c**) Comparing the solubility at 25 °C and permeability of insulin through TR146 cells layers, of insulin in the presence of various concentrations of arginine.

Insulin permeation results in the presence of basic amino acids are expatiated in Figs 1,2 and 3 for arginine, lysine and histidine respectively. The coefficient of apparent permeability of insulin through the actual cell layers, without the interference of the membrane Pcell, was calculated using equation 5 and results are presented in Supplementary Table S2. Amino acid concentrations that showed significant enhancement in insulin permeability include 10, 100 and 200 μg/mL for lysine (Fig. 2b) and 10 μg/mL for histidine (Fig. 3b). Arginine was not able to significantly enhance insulin permeability above that of the control (Fig. 1b). This observation could be accounted for by the inability of arginine to significantly alter the solubility (and therefore the dissociation), of insulin above the control (Supplementary Fig. S1). Arginine has a basic side chain with pKa 12.48 and at lower arginine concentrations of 10–100 μg/mL (solution pH 7.4–7.8), both insulin and arginine would be expected to be ionised and able to form stable unionised ion pairs that would enhance insulin permeability^11^. Significant increase in permeability at these lower concentrations of arginine was not observed possibly due to the presence of a limited concentration of ionised arginine molecules to pair with the ionised insulin moieties. However, as arginine concentration increased, solution pH increased steadily to a maximum of 9.36 at 600 μg/mL arginine. As this pH is close to the isoelectric point of arginine (10.71), it is possible that although insulin molecules were ionised, less ionisation of the arginine molecule occurred, with the presence of fewer counter ions available to form neutral insulin-arginine moieties, and the final result of insignificant difference in insulin permeation was observed^4^,^9^.

From Supplementary Fig. S1, insulin solubility (and therefore insulin ionisation) was observed to significantly increase for lysine concentrations ≥200 μg/mL, with resultant increase in pH from 7 to 9. Thus, at pH of 7 (100 μg/mL lysine), both lysine and insulin molecules would be ionised, the potential for formation of insulin-lysine neutral molecules would be enhanced with a further increase in permeability. As discussed for arginine earlier, concentrations of lysine above 200 μg/mL, pH of the solution moved closer to the isoelectric point of lysine (9.74), causing a reduction in lysine ionisation, a fall in the concentration of lysine counter ions available for neutral complex formation with insulin, and a resultant fall in permeability as shown in Fig. 2c. However at 50 μg/mL lysine, a fall in insulin permeability was observed, probably due to the low concentrations of lysine available for complexation with ionised insulin moieties. Surprisingly, 10 μg/mL lysine significantly enhanced insulin permeability above the control. This observation could be related to the high ionisation of lysine and insulin at pH 7. On the other hand, insulin permeability could have been facilitated in the presence of lysine through amino acid membrane transporters which could have been saturated at higher concentrations^11^,^31^. Insulin permeability results with lysine as permeation enhancer reinforced the fact that for permeation enhancement to occur in dynamic systems, a stringent balance between ionised and non-ionised species must be maintained^4^,^9^,^11^. 100–200 μg/mL was found to be the ideal concentration of lysine to enhance solubility and permeability of insulin *in vitro*.

All concentrations of histidine above 10 μg/mL showed no significant effect on insulin permeation. As highlighted in Supplementary Fig. S1, increasing histidine concentration above 10 μg/mL had no significant effect on insulin solubility in HBSS, with no change in solution pH at all concentrations tested. It was expected that histidine and insulin were ionised at this pH, the availability of counter ions facilitated the formation of insulin-histidine neutral complexes that could enhance insulin permeability significantly. Figure 3c compares the solubility and permeability of insulin at different concentrations of histidine. From here, it can be seen that no advantage was produced for both insulin solubility and permeability when histidine concentration was increased above 10 μg/mL. Similar to results obtained from lysine, 10 μg/mL histidine was able to produce the optimal balance between ionised and non-ionised insulin molecules in solution, as well as possibly effectively triggering peptide transport proteins to enhance the permeation of insulin across TR146 buccal cells.

#### Acidic amino acids

**Figure 4:**
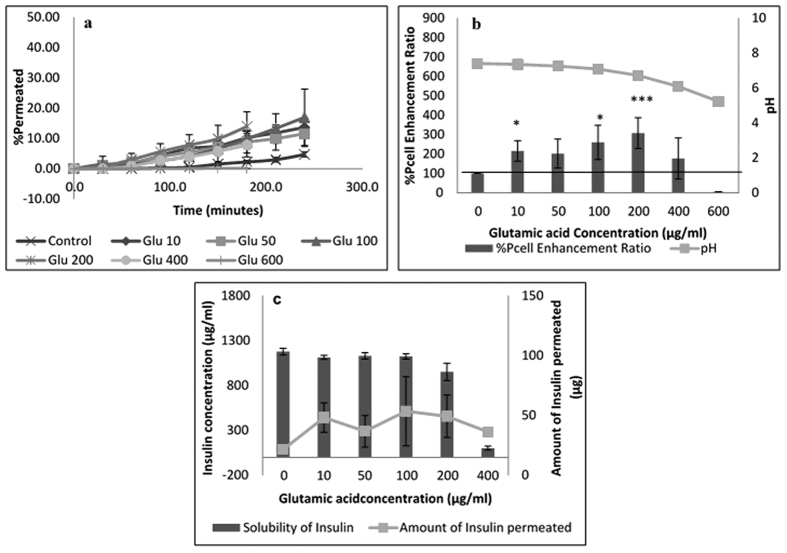
Permeation of human insulin across TR146 buccal cell layers in the presence of increasing glutamic acid concentrations. (**a**) Percentage of insulin permeated in the presence of various concentrations of glutamic acid. (**b**) Comparing the percent enhancement of insulin permeability through TR146 cell layers in the presence of various concentrations of glutamic acid and solution pH. (**c**) Comparing the solubility at 25 °C and permeability of insulin through TR146 cells layers, of insulin in the presence of various concentrations of glutamic acid.

**Figure 5:**
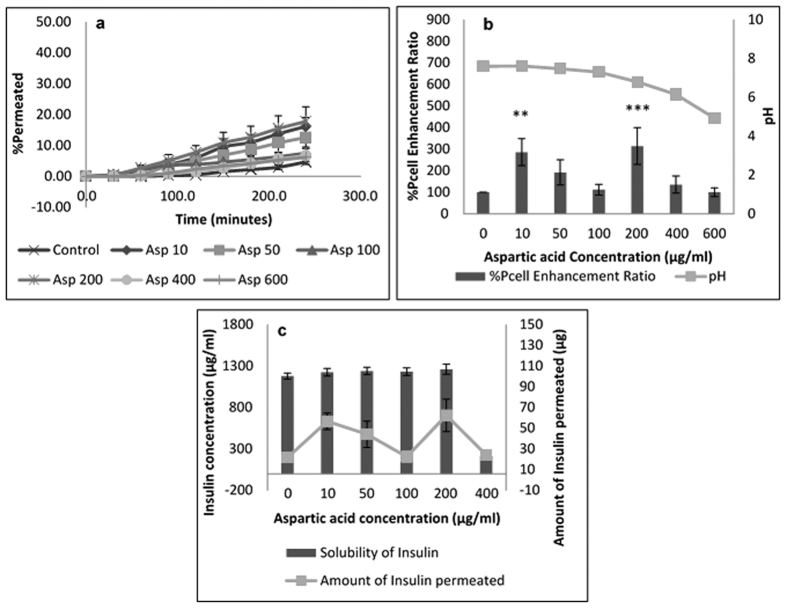
Permeation of human insulin across TR146 buccal cell layers in the presence of increasing aspartic acid concentrations. (**a**) Percentage of insulin permeated in the presence of various concentrations of aspartic acid. (**b**) Comparing the percent enhancement of insulin permeability through TR146 cell layers in the presence of various concentrations of arginine and solution pH. (**c**) Comparing the solubility at 25 °C and permeability of insulin through TR146 cells layers, of insulin in the presence of various concentrations of aspartic acid.

Insulin permeation results in the presence of acidic amino acids are expatiated in Figs 4 and 5 for glutamic acid and aspartic acid respectively. Amino acid concentrations that showed significant enhancement in insulin permeability include 10, 100 & 200 μg/mL for glutamic acid (Fig. 4b) and 10 & 200 μg/mL for aspartic acid (Fig. 5b). These observations for glutamic acid can be directly related to the solubility profile presented in Supplementary Fig. S2 and were expected based on the discussions in sections above. No significant difference in insulin solubility and solution pH was observed with increasing glutamic acid concentrations up to 200 μg/mL, however, at 400 μg/mL, insulin solubility reduced drastically with a solution pH of 6.09 at 600 μg/mL (Fig. 4b). As these pH values were close to the isoelectric point of insulin, less insulin was found in solution with outright precipitation of insulin expected at 600 μg/mL. Thus no insulin was observed to permeate the cells at this high glutamic acid concentration (Fig. 4c); because only the soluble form of a drug is available to permeate cells and be absorbed into the bloodstream^4^,^9^,^32^.

Increasing aspartic acid concentration above 400 μg/mL did not enhance insulin absorption. Similar to results observed for glutamic acid, increasing aspartic acid concentrations to 200 μg/mL did not significantly change the solubility of insulin or solution pH (Supplementary Fig. S2). However for concentrations above 200 μg/mL, insulin permeability drastically declined, with a significant drop in solution pH. However, at all concentrations tested, insulin solubility was higher for aspartic acid than for glutamic acid. This could be attributed to the fact that the pKa of aspartic acid is lower than that of glutamic acid, and thus there was a larger difference in pKa between insulin and aspartic acid, than insulin and glutamic acid. Elshaer *et al*.^7^ suggested that for candidates to sufficiently ionise to produce counter ions that could form non-ionised complexes that enhance permeability, the difference in pKa should be up to 3 units.

#### Safe and effective amino acids as permeation enhancers

Since an aim of this study was to discover effective and safe penetration enhancers for insulin through buccal cells, transepithelial electrical resistance (TEER) of the cell layers (results reported in Supplementary Table S3) and the MTT assay (results represented in Supplementary Fig. S4) were conducted to assess the toxicity of amino acids to the cells, to determine if any observed permeation enhancement was due to destruction of cell layers or increase in cellular toxicity. The TEER results for arginine exhibited no significant reduction in TEER values across all concentrations of arginine used. Thus, cell layer integrity can be said to have been maintained throughout the 4 hours of the permeability experiment^11^,^21^,^33^. Elshaer^11^ reported similar findings where high concentrations of arginine did not significantly affect the TEER measurements before and after permeability studies using Caco-2 cells. This was an expected result since MTT results revealed a reduction in cell viability for concentrations greater than 500 μg/mL. Thus, although arginine was a safe enhancer, it did not significantly enhance the permeation of insulin through TR146 cells^34^. These MTT results for arginine were in conflict with findings reported by Xue *et al*.^21^, where 10–50 μg/mL arginine solutions were found to be toxic to TR146 cells using the MTT assay. This conflict could be explained on the basis that in the current study, TR146 cells were exposed to amino acids for 4 hours (duration of a permeability study^35^) while in the earlier study, exposure times ranged from 24–48 hours. However, because the TR146 cells used for permeability studies formed multiple layers of stratified squamous epithelial cells, while MTT assays were carried out on single cells in active exponential growth phase, low toxicity is to be expected from the permeability studies^21^. TEER results presented in Supplementary Table S3 revealed that 100 μg/mL lysine significantly reduced TEER values post permeability assay, thus the enhancement recorded for this concentration could have been produced by leakage through disrupted cellular layers^21^. MTT assay results (see Supplementary Fig. S4) further revealed a significant concentration dependent decrease in TR146 cell viability with lysine concentrations ≥100 μg/mL which could affect cell integrity^34^. Furthermore, above 100 μg/mL lysine, pH values increased beyond physiologically accepted pH (Fig. 2b). TEER results for histidine (Supplementary Table S3) showed that only 100 μg/mL histidine significantly disrupted cellular integrity, and results from MTT assay (Supplementary Fig. S4) revealed that histidine was non-toxic to TR146 cells even at the highest concentration (800 μg/mL) tested. Therefore for the basic amino acids, although 10, 100 and 200 μg/mL lysine and 10 μg/mL histidine were effective as permeation enhancers for insulin, only 10 μg/mL of each amino acid were both effective and non-toxic to TR146 cells (Supplementary Fig. S5) and were chosen as optimal concentrations that effectively enhanced permeation of insulin without affecting cell viability or causing membrane disruption^36^,^37^,^38^. 10 μg/mL histidine showed significantly higher permeation enhancement than 10 μg/mL lysine.

TEER results from Supplementary Table S3 showed no significant reduction in TEER values at all concentrations of glutamic acid tested, and only 600 μg/mL aspartic acid significantly reduced TEER values. While MTT assay results (Supplementary Fig. S4) revealed toxicity of cells only at 800 μg/mL for both glutamic and aspartic acids respectively. However these concentrations were not employed in permeability studies. Thus, glutamic and aspartic acids was found to be safe and non-toxic to cells across all tested concentrations. Thus, the enhancement in insulin permeability by 10, 100 & 200 μg/mL glutamic acid and 10 & 200 μg/mL aspartic acid could be attributed solely to the amino acid facilitation of permeation through the formation of neutral ion pairs with insulin, or through the use of membrane peptide transporters, rather than disruption of cell layers or reduced cellular integrity^9^,^11^. Therefore for the acidic amino acids 10, 100 and 200 μg/mL glutamic acid and 10 & 200 μg/mL aspartic acid were able to effectively and safely enhance insulin permeation without any toxicity to TR146 cells (Supplementary Fig. S6).

#### Sodium deoxycholate

**Figure 6:**
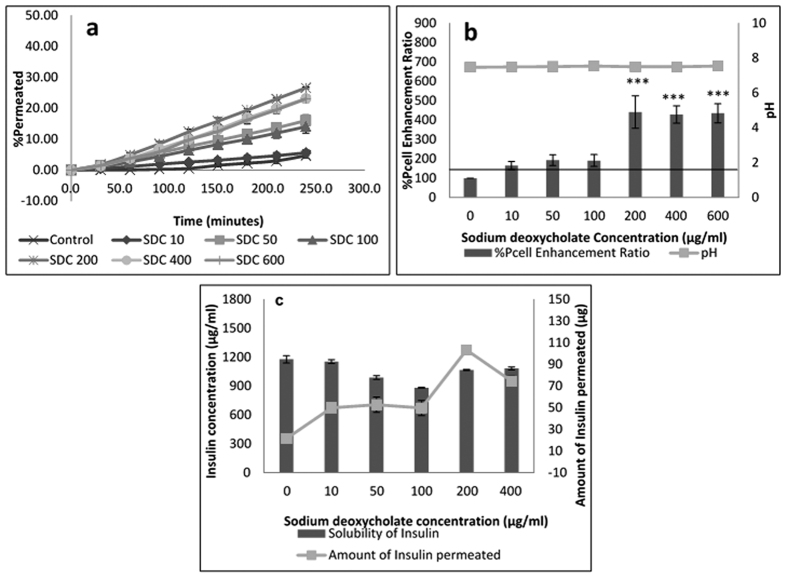
Permeation of human insulin across TR146 buccal cell layers in the presence of increasing sodium deoxycholate concentrations. (**a**) Percentage of insulin permeated in the presence of various concentrations of sodium deoxycholate. (**b**) Comparing the percent enhancement of insulin permeability through TR146 cell layers in the presence of various concentrations of aspartic acid and solution pH. (**c**) Comparing the solubility at 25 °C and permeability of insulin through TR146 cells layers, of insulin in the presence of various concentrations of sodium deoxycholate.

Sodium deoxycholate (SDC) is a bile acid salt with pKa 6.58 (Supplementary Table S1) which has been widely investigated for its role in enhancing permeation of drugs across cell membranes^39^,^40^,^41^,^42^. In this study, the effectiveness and safety of the amino acids as permeation enhancers were compared against that obtained with sodium deoxycholate, to verify if amino acids could serve as less toxic and safer alternative permeation enhancers for insulin than bile salts. From Fig. 6b, 200, 400 and 600 μg/mL sodium deoxycholate were able to significantly enhance insulin permeability. The high enhancement with SDC was an expected finding based on the ability of surfactants to fluidise the lipid membranes of the cells and impact on their integrity and barrier forming properties^39^. This effect was seen with TEER results, where a significant linear drop in TEER values signifying a reduction in cell layer integrity was recorded at concentrations of SDC > 100 μg/mL (Supplementary Table S2). Cell viability results (Supplementary Fig. S4) further confirmed increased toxicity of cells exposed to 100 μg/mL SDC and above. Xue *et al*.^21^ reported no significant change in TEER results in the presence of 10 and 50 μg/mL Na deoxycholate, however 10–50 μg/mL reduced the viability of TR146 cells after 24–48 hour exposure. Because the increase in insulin permeability afforded by SDC showed a linear trend (Fig. 6c), while that of the amino acids showed a somewhat “zig-zag trend”, it is possible that for the amino acids, a further mechanism (such as triggering of amino acid/peptide transporters) outside disruption of cell membranes, came into play. Surfactants such as SDC enhance drug permeation paracellularly by binding to calcium ions or disruption of hemi desmosomes between cells which might enhance both paracellular and transcellular transport^43^,^44^. Thus, because of the toxicity exhibited by SDC, none of the tested concentrations yielded effective and safe permeation enhancement.

Supplementary Fig. S7 compares the concentrations of enhancers that effectively and safely improved insulin permeation across TR146 cell layers *in vivo*. It can be noted that apart from arginine which showed no significant improvement in the coefficient of apparent permeability through the cell layers (Pcell), at 10 μg/mL all amino acids were safe and effective; SDC was safe but ineffective. At high concentrations of 200 μg/mL, the acidic amino acids were both safe and effective while lysine and SDC were effective but unsafe; and at 100 μg/mL, only glutamic acid was safe and effective; lysine was effective but unsafe, while SDC was safe but ineffective. Thus, we have been able to demonstrate that acidic and basic amino acids act as effective penetration enhancers which circumvent the toxicity issues related with the use of surfactants such as SDC.

### Route of insulin transport

**Figure 7:**
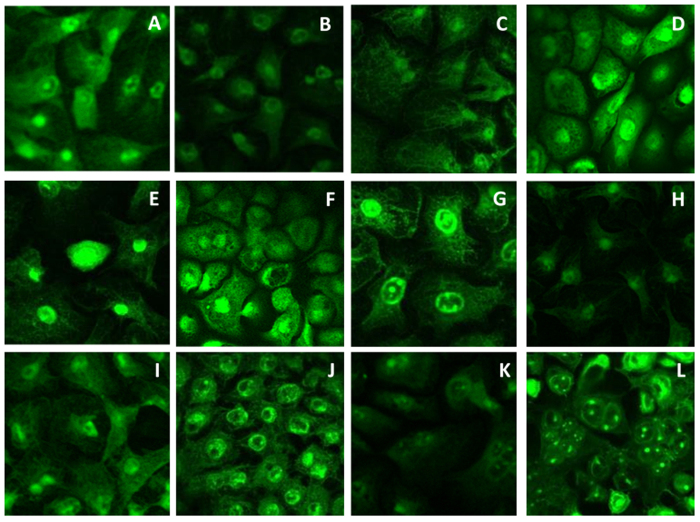
Confocal microscopic images showing transport pathway of FITC-labelled insulin through TR146 buccal cell layers in the presence of amino acid concentrations that effectively enhanced insulin permeability. Insulin observed to traverse transcellularly with localisation in the nucleus. Control (**A**), Arg 50 μg/ml (**B**), Asp10 μg/ml (**C**), Asp 200 μg/ml (**D**), Glu 10 μg/ml (**E**), Glu 100 μg/ml (**F**), Glu 200 μg/ml (**G**), Hist 10 μg/ml (**H**), Lys 10 μg/ml (**I**), Lys 100 μg/ml (**J**), Lys 200 μg/ml (**K**), Na 200 μg/ml (**L**).

After identifying safe and effective amino acid permeation enhancers for insulin, the next priority was to determine the route of insulin transport (whether paracellular or trascellular) across the buccal cell layers. In order to test our first hypothesis that formation of neutral insulin-amino acid ion pairs with higher lipophilicity would enhance transcellular insulin transport by passive diffusion, FITC-labelled insulin (6 μg/mL) was transported for 4 hours across TR146 cells grown on transwell inserts and analysed by confocal microscopy. Results revealed localisation of FITC-insulin molecules in the cell cytoplasm (not paracellularly), both in the presence and absence of amino acids and bile salts (Fig. 7). Supplementary Fig. S8 shows DAPI-stained images confirming that FITC-insulin localisation occurred close to the nucleus. These findings were in contrast to investigations for FITC-labelled dextran 4 kDa as a marker for paracellular transport via TR146 cell layers; which traversed the buccal cells via the paracellular route with no localisation at all in the nucleus or cytoplasm (Supplementary Fig. S9). These results confirmed our hypothesis that insulin transport did not occur paracellularly but was through the transcellular route. The paracellular route has been reported to transport small hydrophilic molecules (≤300 Da) by passive diffusion. The absence of paracellular transport for insulin was expected due to its large molecular weight (5,800 Da) and the fact that the intercellular spaces of the buccal cavity are highly lipophilic due to the extrusion of the lipid content of the membrane coating granules that occur in the upper two-thirds of the buccal epithelium into the intercellular spaces, thereby inhibiting buccal paracellular transport^39^,^40^. The transcellular transport observed in the absence of amino acids (control) indicates that insulin transport possibly occurred via insulin receptors. Similar findings were recorded by Thompson *et al*.^45^ who investigated the transport of FITC-insulin across Caco-2 cell lines (model for small intestines) and reported localisation of FITC-insulin in the cell cytoplasm after 30 minutes exposure at 3 μg/mL. This uptake of insulin was reported to be mediated by interactions with insulin receptors^45^. Xu^46^ reported that confocal microscopic imaging of FITC-labelled insulin showed that insulin could be transported via both the transcellular and paracellular routes in rabbit buccal mucosa.

### Mechanism of insulin transport through TR146 cell culture model

Upon confirming that insulin traversed the buccal cell layers via the transcellular and not the paracellular route, we needed to test our second hypothesis that this transport was by active transporter facilitated mechanisms. Glutamic acid was chosen as a representative amino acid since it showed enhanced permeation and overall safety over the widest range of concentrations tested. Similarly, Sauberlich^47^ reported that of all the 20 amino acids fed in excess of recommended doses to weanling rats, L-glutamic acid showed the least toxicity expressed as inhibition of growth, and showed the least absorption into the plasma.

**Figure 8:**
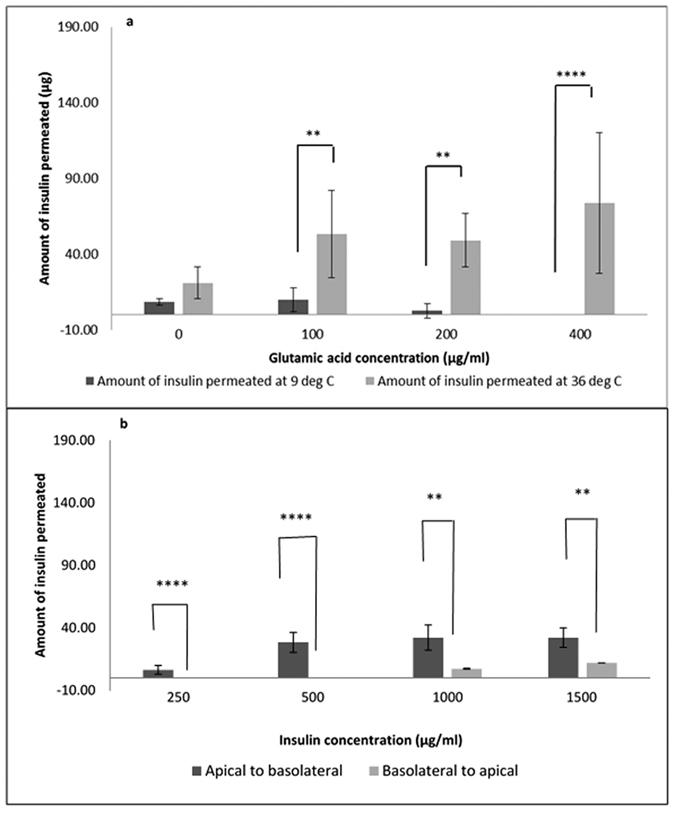
To investigate active insulin transport in the presence of amino acids. (**a**) Insulin concentration was fixed at 1mg/ml while glutamic acid concentration was varied, and permeation experiments carried out at 9 °C for 4 hours. (**b**) Vectoral transport of increasing concentrations of insulin with glutamic acid concentration was fixed at 200 μg/ml. Temperature and concentration dependent fall in insulin permeation at suboptimal temperatures, and lower basal-to-apical insulin transport, confirm the presence of active transport of insulin.

To confirm the presence of active transport, the concentration of insulin was kept constant at 1 mg/mL while glutamic acid concentration was varied at 0, 100, 200 and 400 μg/mL. Transport experiments were carried out in a cold room at 9 °C. Results depicted in Fig. 8a revealed a significant reduction in insulin permeation at 9 °C compared with 36 °C. Cellular activities cease at suboptimal temperatures, therefore a fall in permeation at lower temperatures would depict a cessation of active transport.

Furthermore, at 9 °C the amount of insulin permeated decreased with increasing amino acid concentration. These results point to the presence of active transport of insulin-glutamic acid neutral complexes formed by ion-pairing, possibly via amino acid/peptide transporters in the cell membranes^48^. Membrane transporters are membrane bound proteins that selectively facilitate the transport of substances across biological membranes^49^. Inactivation of enzymatic activity at suboptimal temperatures results in reduced function of these transporters, thereby inhibiting insulin permeation across the cell layer. Furthermore, as transporter activity had already been compromised by the low temperature, increasing amino acid concentrations above 100 μg/mL caused a further reduction and ultimate cessation of insulin transportation probably through early saturation of the transporters^11^. It is possible that the low transport recorded at 400 μg/mL glutamic acid could be related to the low solubility of insulin at this concentration (Supplementary Fig. S2), however this concentration showed insulin permeation at 36 °C (Fig. 4c), thereby suggesting that the change was due to saturation of the transporter proteins^11^,^50^.

To further establish the presence of active transport in this system, glutamic acid concentration was maintained at 200 μg/mL while insulin concentration was varied at 250, 500, 1000 and 1500 μg/mL (Fig. 8b). It was observed that increasing insulin concentrations above 500 μg/mL showed no significant increase in permeation. Biochemical reports on the buccal epithelium reveal the extrusion of lipid components of the epithelium into the paracellular spaces, thereby increasing its lipophilicity and causing an added permeation barrier^37^. Thus, passive paracellular diffusion would not be expected for a macro molecule such as insulin (5,800 Da) since this route favours the transport of hydrophilic molecules ≤300 Da^48^. Passive diffusion relies on the concentration gradient between the extracellular and intracellular compartments, while active transport can occur against a concentration gradient, therefore, no increase in permeation would be observed with increasing drug concentration if the molecule was solely transported actively^48^. The rate of transport in passive diffusion is governed by Fick’s law and is therefore directly dependent on the initial concentration; with active transport, this relationship is only relevant at low concentrations, and with increasing concentrations, carrier saturation occurs and no increase in uptake is observed with increasing concentrations^48^. Thus results from Fig. 8b, confirm the presence of active transcellular transport for insulin. We further investigated vectoral transport of insulin across the cell layers (Fig. 8b). Reversal of transport direction from apical-basolateral, to basolateral-apical revealed a concentration dependent decrease in insulin permeation, with no permeation recorded at 250 and 500 μg/mL insulin. According to Biopharmaceutical Classification System guidelines, to confirm the presence of passive transport *in vitro*, there should be a lack of dependence on initial concentration and directional transport; since the concentration of nutrient transporter proteins are higher at the apical surface than at the basolateral surface.^48^. Thus these results further point to the presence of active transport for insulin. Artusson *et al*.^50^ agreed that highly potent hydrophilic compounds such as peptide antigens, with similar structures as food nutrients could be transported across cell layers by a combination of active facilitated transport as well as passive transport. However, these results obtained for insulin are the first indication of a possible amino acid mediated transport of insulin via formation of insulin-amino acid neutral complexes by the ion pairing mechanism.

## Conclusion

The results of these studies demonstrated a “bell-shaped” impact on insulin permeability upon increasing the concentration of amino acid counter ions, and suggest that the concentration of the counter ion requires optimisation to provide a balance between insulin dissociation and permeation. Interestingly, investigating the mechanism and route of insulin permeation using increasing concentrations of insulin at suboptimal temperatures, reversed transport direction and confocal microscopy revealed that insulin was transported by passive and active transcellular processes provided by the presence of insulin receptors and amino acid nutrient transporters on the cell membrane. Therefore, amino acids were established as safer, effective and novel penetration enhancers, than the commonly used surfactant Na deoxycholate, for insulin using the ion-pairing mechanism.

## Materials and Methods

### Materials

TR146 cell line was purchased from Public Health England, UK. Human recombinant insulin, L-arginine (non-animal source, cell culture tested), L-lysine (≥97%), L-histidine (≥Reagent Plus 99%), L-glutamic acid (minimum 99% TLC), L-aspartic acid (99%), sodium deoxycholate, thiazolyl blue tetrazolium bromide (methylthiazolydiphenyl-tetrazolium bromide; ≥97%; enzymatic), dimethyl sulfoxide (DMSO) and trifluoroacetic acid were purchased from Sigma-Aldrich, UK. Hanks balanced salt solution (HBSS), Fetal bovine saline (FBS) were obtained from Gibco Lab., UK. Acetoniltrile and absolute ethanol were purchased from Fisher scientific, UK. Hank’s balanced salt solution (HBSS), fetal bovine serum (FBS), Hams F-12 nutrient mix and trypsin-EDTA were obtained from Gibco^®^ Lab., UK. Gentamicin and penicillin/ streptomycin were obtained from Bio Sera, UK. Acetoniltrile and absolute ethanol were purchased from Fisher scientific, UK. 6 well plates, 6 well polycarbonate transwell inserts and 12 well polyester transwell inserts were purchased from Appletonwoods, UK. All the chemicals and reagents were used as obtained. All water used was double-distilled and autoclaved.

### Methods

#### High Performance Liquid Chromatography (HPLC) Assay

Quantitative analysis of insulin in solution was achieved by gradient RP-HPLC adapted from Sarmento^51^. A Waters HPLC system (Alliance) with gradient pump and UV/fluorescent detector was used, employing a reversed phase RP-C18 analytical column (Phenomenex 110A, 150 × 4.6 mm, 5 μm). The mobile phase consisted of solution A: 0.1% v/v TFA in water and solution B: absolute acetonitrile both filtered under vacuum and sonicated prior to use. A gentle gradient was run from 74:26 to 67:33 compositions of A:B over 3 mins, and then maintained at 67:33 for 4 mins. Pump flow rate was 1 mL/min, with sample injection volume of 10 μL and UV detector wavelength of 214 nm. Column and samples were maintained at room temperature. The maximum wavelength of absorption of insulin had been pre-determined using a Unicam UV-visible spectrophotometer. The method was validated using ICH guidelines.

#### Insulin solubility in water and HBSS

Solubility studies were carried out at 25 °C by adding excess amounts of insulin in 5 ml of deionised water containing 4, 27 or 50 μg/mL concentrations of arginine and glutamic acid respectively. pH of the solutions was measured using a pHenomenal pH meter (VWR-USA). The solutions were then allowed to stir on a multi IKA magnetic stirrer overnight^7^,^52^. After standing for 2 hours, the supernatant was filtered through 0.45 μm filters, and insulin content quantified by HPLC. Similar experiments were carried out using 10, 50, 100, 200, & 400 μg/mL concentrations of basic (arginine, lysine & histidine) and acidic (glutamic acid & aspartic acid) amino acids and Na deoxycholate, using HBSS in place of deionised water. This was because *in vitro* permeability studies would be carried out in HBSS. Solutions without amino acids were used as control.

#### TR146 Cell Culture Procedures

TR146 cells were maintained in 75 cm^3^ T-flasks in Ham’s F-12 cell culture media fortified with 10% FBS, 2 mM glutamine, 100 IU penicillin/streptomycin, 10 μg gentamicin; and incubated at 37 °C, 5% CO_2_ and 95% air. Media change occurred every 2–3 days and at 90% confluence, cells were passaged using 5 mL trypsin-EDTA solution, and seeded unto 12 well transwell inserts at a density of 24,000 cells/cm^2^. Transepithelial electrical resistance (TEER) was used to monitor cellular layer integrity 30 minutes after each media change^11^. Passage numbers 30–39 were used for these experiments.

#### Trans-epithelium electric resistance (TEER)

The ohmic resistance (resistance to current flow via the paracellular pathway) of cells grown on transwell inserts was measured using an EVOM volt ohmmeter with chopstick electrodes. The electrodes were placed erect, such that the longer arm just touched the fluid in the basolateral chamber, while the shorter arm barely touched the apical membrane. TEER which reveals the integrity of the cellular layers was calculated using the equation below, and reported as mean ± standard deviation of triple readings from replicate transwells (n = 9).


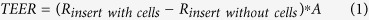


where R is the measured resistance and A is the cross-sectional area of the cells.

#### Cytotoxicity testing via MTT assay

The methylthiazolydiphenyl-tetrazolium bromide (MTT) assay was used to assess the cytotoxicity of the enhancers on TR146 cells in their exponential growth phase. Cells were seeded unto 96 well plates at a density of 24,000 cells/cm^2^ and incubated for 24 hours. The media was removed and 200 μL of increasing concentrations (25, 50, 100, 200, 400 and 800 μg/mL) of each enhancer in HBSS was added to each well in replicates of 12 per concentration. Cells were incubated for 4 hours (the duration of permeability experiments) after which 20 μL MTT solution (5 mg/mL in PBS) was added to each well and incubated for a further 4 hours. Finally, wells were emptied and solutions replaced with 100 μL dimethyl sulfoxide (DMSO) and left to shake at 140 rpm and 37 °C for 30 minutes. The absorbance was read at 490 nm using a Multiskan spectrum plate reader (Thermoscientific, UK). The relative enzyme reactivity and therefore cell viability was calculated from:


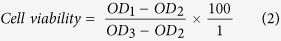


OD_1_, OD_2_ and OD_3_ are the optical densities of wells with cells and enhancer solution, blank wells without cells or enhancer solution, and wells with cells but no enhancer solution respectively.

#### *In vitro* transbuccal permeation studies

Permeability studies were conducted as described by Nielsen and Rassing^53^ at 36 °C and 140 rpm in an orbital plate shaker. Test solutions containing 1000 μg/mL insulin dissolved in HBSS in the presence of increasing concentrations (0,10, 50, 100, 200, 400 and 600 μg/mL) of amino acids as well as sodium deoxycholate were prepared and stirred until a clear solution was observed, and pH of solutions measured. Cells on transwell inserts were rinsed twice with HBSS (37 °C) by adding 0.5 mL and 1.5 mL respectively to the apical and basolateral chambers respectively. After incubation for 30 mins, TEER readings were taken in triplicate per insert. The basolateral chamber was then replaced with fresh HBSS and the apical chamber with 0.5 mL test solution. Immediately, 100 μL of test solution was withdrawn from the apical and basolateral chambers at time 0. Basolateral sampling of 700 μL was done every 30 mins for 4 hours with immediate replacement with pre-warmed HBSS. At the end of the experiment, 100 μL of apical solution was taken. Test solutions without permeation enhancers were used as control^21^,^54^,^55^. After 4 hours, cells were rinsed twice with 0.5 mL HBSS and equilibrated for 30 minutes after which TEER values were recorded. All samples were made up to 700 μL where necessary, using HBSS, and analysed for insulin content by HPLC, and resultant data used to calculate the apparent coefficient of permeability through the insert with cells (P_app_), the cell-free apparent permeability coefficient (P_ins_), the cell apparent permeability coefficient (P_cell_), the percent enhancement ratio (%ER) and the recovery %.


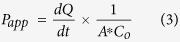



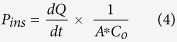



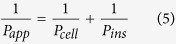



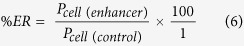



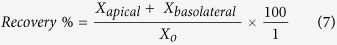


dQ/dt is the rate of insulin transport at steady state in μg/s, A is the cross sectional area of cells in cm^2^, C_O_ is the initial apical solution concentration in μg/mL, X_apical_ is the quantity of insulin remaining in the apical chamber after 4 hours, X_basolateral_ is the cumulative amount of insulin sampled from the basolateral chamber after 4 hours and X_o_ is the initial quantity of insulin in the apical chamber at the start of the experiment^21^,^55^,^56^.

Insulin stability after permeation was monitored by HPLC for changes in retention time and/or presence of multiple peaks on the HPLC chromatogram.

#### Route of insulin transport

Solutions containing FITC-labelled insulin (0.6 mg/mL in HBSS) along with concentrations of enhancers that effectively enhanced insulin permeability, were incubated with TR146 cells; as with the transport experiments^57^. TEER values in HBSS were measured before and after the experiment. After 4 hours, transwell inserts with cells were washed three times with warm HBSS and fixed with 4% paraformaldehyde (PFA) in PBS for 10 minutes, and cells were rinsed four times with PBS. Inserts were placed on microscope slides and the membrane carefully cut out using a scalpel. Two drops of DAPI (4′6′-diamidino-2-phenylindole) in Vectashield (Vector Laboratories,UK) were used to mount the specimen which was immediately covered with a microscope cover slip and allowed to air dry for 10 minutes. Samples were then imaged using a Leica laser confocal scanning microscope (Germany) at 10x (dry) and 40x (oil) magnifications.

#### Mechanism of insulin transport

To investigate the presence of passive transport, permeability studies described above were carried out at 36 °C (optimised temperature for TR146 cells) using a single concentration of glutamic acid (200 μg/mL) with increasing concentrations of insulin (250, 500, 1000 and 1500 μg/mL) and all permeability indices calculated. While to investigate the presence of active amino acid-facilitated transport of insulin, permeability studies were repeated at suboptimal temperatures (9 °C) with 1000 μg/mL insulin in the presence of increasing concentrations of glutamic acid (100, 200 &, 400 μg/mL). Transwells were maintained at 9 °C for 1 hour prior to commencing studies to ensure a cessation of enzymatic activity, and the resulting insulin transport analysed by HPLC. Cellular activities cease at suboptimal temperatures, therefore a fall in permeation at lower temperatures would depict a cessation of active transport.

#### Statistical Analysis

All data was presented as mean ± standard deviation and analysed using One-way ANOVA and Turkey-Kramar multiple comparison post-test from Graphpad Prism version 6.07 (California, USA). Level of significance was quoted as p < 0.05 (probability values of 95%).

## Additional Information

**How to cite this article**: Iyire, A. *et al*. Pre-formulation and systematic evaluation of amino acid assisted permeability of insulin across *in vitro* buccal cell layers. *Sci. Rep.* **6**, 32498; doi: 10.1038/srep32498 (2016).

## Supplementary Material

Supplementary Information

## References

[b1] Owens D. R., Zinman B. & Bolli G. Alternative routes of insulin delivery. Diabetic Med. 20, 886–898 (2003). 14632713 10.1046/j.1464-5491.2003.01076.x

[b2] Veuillez F., Kalia Y. N., Jacques Y., Deshusses J. & Buri P. Factors and strategies for improving buccal absorption of peptides. Eur. J. Pharm. Biopharm. 51, 93–109, doi: 10.1016/S0939-6411(00)00144-2 (2001). 11226816

[b3] Milstein S. J. . Partially unfolded proteins efficiently penetrate cell membranes—implications for oral drug delivery. J. Controlled Release 53, 259–267 (1998). 10.1016/s0168-3659(97)00259-9 9741933

[b4] Samiei N. . Ion-pair strategy for enabling amifostine oral absorption: rat *in situ* and *in vivo* experiments. Eur. J. Pharm. Sci. 49, 499–504 (2013). 23643735 10.1016/j.ejps.2013.04.025

[b5] Miller J. M. *The impact of molecular complexation on intestinal membrane permeation*. The University of Michigan (2009).

[b6] Hemenway J. N. . Preparation and physicochemical characterization of a novel water‐soluble prodrug of carbamazepine. J. Pharm. Sci. 99, 1810–1825 (2010). 19774656 10.1002/jps.21952

[b7] ElShaer A., Khan S., Perumal D., Hanson P. & R Mohammed A. Use of amino acids as counterions improves the solubility of the BCS II model drug, indomethacin. Curr. Drug Deliv. 8, 363–372 (2011). 21453261 10.2174/156720111795767924

[b8] Ivaturi V. D. & Kim S. K. Enhanced permeation of methotrexate *in vitro* by ion pair formation with L‐arginine. J. Pharm. Sci. 98, 3633–3639 (2009). 19117046 10.1002/jps.21663

[b9] Samiei N., Shafaati A., Zarghi A., Moghimi H. & Foroutan S. Enhancement and *in vitro* evaluation of amifostine permeation through artificial membrane (PAMPA) via ion pairing approach and mechanistic selection of its optimal counter ion. Eur. J Pharm. Sci. 51, 218–223 (2014). 24161609 10.1016/j.ejps.2013.10.002

[b10] Chen B. L., Wu X., Babuka S. J. & Hora M. Solubility of recombinant human tissue factor pathway inhibitor. J. Pharm. Sci. 88, 881–888 (1999). 10479349 10.1021/js9900708

[b11] ElShaer A., Hanson P. & R Mohammed A. A novel concentration dependent amino acid ion pair strategy to mediate drug permeation using indomethacin as a model insoluble drug. Eur J. Pharm. Sci. 62, 124–131 (2014). 24907680 10.1016/j.ejps.2014.05.022

[b12] Ohshima H. & Makino K. Colloid and Interface Science in Pharmaceutical Research and Development. (Elsevier, 2014).

[b13] Matsuura J., Powers M. E., Manning M. C. & Shefter E. Structure and Stability of Insulin Dissolved in 1-Octanol. J. Am. Chem. Soc. 115, 1261–1264, doi: 10.1021/Ja00057a006 (1993).

[b14] Hames D. & Hooper N. Biochemistry, 3 th. New York, Taylor and Francis (2005).

[b15] Xu X., Fu Y., Hu H., Duan Y. & Zhang Z. Quantitative determination of insulin entrapment efficiency in triblock copolymeric nanoparticles by high-performance liquid chromatography. J. Pharm. Bio. Analysis 41, 266–273, doi: 10.1016/j.jpba.2005.10.016 (2006). 16303273

[b16] British Pharmacopoeia Vol. 1. London. *The British Pharmacopoeia Commission* (2012).

[b17] Lens J. The solubility curve and the purity of insulin. J. Biol. Chem. 164, 223–231 (1946). 20989483

[b18] Kramer R. M., Shende V. R., Motl N., Pace C. N. & Scholtz J. M. Toward a molecular understanding of protein solubility: increased negative surface charge correlates with increased solubility. Biophys. J. 102, 1907–1915 (2012). 22768947 10.1016/j.bpj.2012.01.060 PMC3328702

[b19] Landreh M. . Insulin solubility transitions by pH‐dependent interactions with proinsulin C‐peptide. FEBS J. 279, 4589–4597 (2012). 23106816 10.1111/febs.12045

[b20] Trevino S. R., Scholtz J. M. & Pace C. N. Measuring and increasing protein solubility. J. Pharm. Sci. 97, 4155–4166 (2008). 18240286 10.1002/jps.21327

[b21] Xue X.-y. . Promoting effects of chemical permeation enhancers on insulin permeation across TR146 cell model of buccal epithelium *in vitro*. Drug Chem. Toxicol. 35, 199–207 (2012). 21848502 10.3109/01480545.2011.589848

[b22] Yin L. . Drug permeability and mucoadhesion properties of thiolated trimethyl chitosan nanoparticles in oral insulin delivery. Biomaterials 30, 5691–5700 (2009). 19615735 10.1016/j.biomaterials.2009.06.055

[b23] Wintersteiner O. & Abramson H. A. The isoelectric point of insulin electrical properties of adsorbed and crystalline insulin. J. Biol. Chem. 99, 741–753 (1933).

[b24] Uchiyama T. . Enhanced Permeability of Insulin across the Rat Intestinal Membrane by Various Absorption Enhancers: Their Intestinal Mucosal Toxicity and Absorption‐enhancing Mechanism of n‐Lauryl‐β‐D‐maltopyranoside. J. Pharm. Pharmacol. 51, 1241–1250 (1999). 10632081 10.1211/0022357991776976

[b25] Türker S., Onur E. & Ózer Y. Nasal route and drug delivery systems. Pharm. World Sci. 26, 137–142 (2004). 15230360 10.1023/b:phar.0000026823.82950.ff

[b26] Yamamoto A., Hayakawa E., Kato Y., Nishiura A. & Lee V. A mechanistic study on enhancement of rectal permeability to insulin in the albino rabbit. J. Pharm. Exp. Ther. 263, 25–31 (1992). 1403789

[b27] Gandhi R. & Robinson J. Mechanisms of penetration enhancement for transbuccal delivery of salicylic acid. Int J Pharm 85, 129–140 (1992).

[b28] Rotunda A. M., Suzuki H., Moy R. L. & Kolodney M. S. Detergent effects of sodium deoxycholate are a major feature of an injectable phosphatidylcholine formulation used for localized fat dissolution. Dermatol. Surg. 30, 1001–1008 (2004). 15209790 10.1111/j.1524-4725.2004.30305.x

[b29] Sun S., Liang N., Kawashima Y., Xia D. & Cui F. Hydrophobic ion pairing of an insulin-sodium deoxycholate complex for oral delivery of insulin. Int. J. Nanomed. 6, 3049 (2011). 10.2147/IJN.S26450 PMC3230571 22162661

[b30] Quintanar-Guerrero D., Allémann E., Fessi H. & Doelker E. Applications of the ion-pair concept to hydrophilic substances with special emphasis on peptides. Pharm. Res. 14, 119–127 (1997). 9090697 10.1023/a:1012076022420

[b31] Bröer S. Amino acid transport across mammalian intestinal and renal epithelia. Physiol. Rev. 88, 249–286 (2008). 18195088 10.1152/physrev.00018.2006

[b32] Dahan A. & Miller J. M. The solubility–permeability interplay and its implications in formulation design and development for poorly soluble drugs. The AAPS Journal 14, 244–251 (2012). 22391790 10.1208/s12248-012-9337-6 PMC3326160

[b33] Nielsen H. M. & Rassing M. R. Nicotine permeability across the buccal TR146 cell culture model and porcine buccal mucosa *in vitro*: effect of pH and concentration. Eur. J. Pharm. Sci. 16, 151–157 (2002). 12128169 10.1016/s0928-0987(02)00083-0

[b34] van Meerloo J., Kaspers G. J. & Cloos J. In Cancer Cell Culture 237–245 (Springer, 2011).

[b35] Jacobsen J., Pedersen M. & Rassing M. R. TR146 cells as a model for human buccal epithelium: II. Optimisation and use of a cellular sensitivity MTS/PMS assay. Int J Pharm 141, 217–225 (1996).

[b36] Khafagy el S., Morishita M., Onuki Y. & Takayama K. Current challenges in non-invasive insulin delivery systems: a comparative review. Adv. Drug. Deliv. Rev. 59, 1521–1546, doi: 10.1016/j.addr.2007.08.019 (2007). 17881081

[b37] Patel V. F., Liu F. & Brown M. B. Advances in oral transmucosal drug delivery. J. Control. Release 153, 106–116 (2011). 21300115 10.1016/j.jconrel.2011.01.027

[b38] Kumria R. & Goomber G. Emerging trends in insulin delivery: Buccal route. J. Diabetol. 2, 1–9 (2011).

[b39] Şenel S. & Hıncal A. A. Drug permeation enhancement via buccal route: possibilities and limitations. J. Control. Release 72, 133–144, doi: 10.1016/S0168-3659(01)00269-3 (2001). 11389992

[b40] Shojaei A. H. Buccal mucosa as a route for systemic drug delivery: a review. J. Pharm. Pharm. Sci. 1, 15–30 (1998). 10942969

[b41] Nicolazzo J. A., Reed B. L. & Finnin B. C. Buccal penetration enhancers—how do they really work? J. Control. Release 105, 1–15 (2005). 15894393 10.1016/j.jconrel.2005.01.024

[b42] Junginger H. E., Hoogstraate J. A. & Verhoef J. C. Recent advances in buccal drug delivery and absorption — *in vitro* and *in vivo* studies. J. Control. Release 62, 149–159, doi: 10.1016/S0168-3659(99)00032-2 (1999). 10518646

[b43] Buchert M., Turksen K. & Hollande F. Methods to examine tight junction physiology in cancer stem cells: TEER, paracellular permeability, and dilution potential measurements. Stem Cell Rev. 8, 1030–1034 (2012). 22134928 10.1007/s12015-011-9334-7

[b44] Moghimipour E., Ameri A. & Handali S. Absorption-Enhancing Effects of Bile Salts. Molecules 20, 14451–14473 (2015). 26266402 10.3390/molecules200814451 PMC6332414

[b45] Thompson C. . Uptake and transport of novel amphiphilic polyelectrolyte-insulin nanocomplexes by Caco-2 cells—towards oral insulin. Pharmaceut. Res. 28, 886–896 (2011). 10.1007/s11095-010-0345-x 21213024

[b46] Xu H.-B. . Hypoglycaemic effect of a novel insulin buccal formulation on rabbits. Pharmacol. Res. 46, 459–467, doi: 10.1016/S1043661802002049 (2002). 12419651

[b47] Sauberlich H. Studies on the toxicity and antagonism of amino acids for weanling rats. The J. Nutr. 75, 61–72 (1961). 13746747 10.1093/jn/75.1.61

[b48] Lehr C.-M. Cell culture models of biological barriers: in vitro test systems for drug absorption and delivery. (CRC Press, 2003).

[b49] Lin L., Yee S. W., Kim R. B. & Giacomini K. M. SLC transporters as therapeutic targets: emerging opportunities. Nat. Rev. Drug Disc. 14(8), 543–560 (2015). 10.1038/nrd4626 PMC4698371 26111766

[b50] Artursson P., Palm K. & Luthman K. Caco-2 monolayers in experimental and theoretical predictions of drug transport. Adv. Drug Del. Rev. 64, Supplement, 280–289, doi: http://dx.doi.org/10.1016/j.addr.2012.09.005 (2012). 10.1016/s0169-409x(00)00128-9 11259831

[b51] Sarmento B., Ribeiro A., Veiga F. & Ferreira D. Development and validation of a rapid reversed‐phase HPLC method for the determination of insulin from nanoparticulate systems. Biomed. Chrom. 20, 898–903 (2006). 10.1002/bmc.616 16389645

[b52] Nicolescu C., Aramă C., Nedelcu A. & Monciu C.-M. Phase solubility studies of the inclusion complexes of repaglinide with β-cyclodextrin and β-cyclodextrin derivatives. Farmacia 58, 620–628 (2010).

[b53] Nielsen H. M. & Rassing M. R. TR146 cells grown on filters as a model of human buccal epithelium: III. Permeability enhancement by different pH values, different osmolality values, and bile salts. Int J Pharm 185, 215–225 (1999). 10460917 10.1016/s0378-5173(99)00165-9

[b54] Portero A., Remuñán-López C. & Nielsen H. M. The potential of chitosan in enhancing peptide and protein absorption across the TR146 cell culture model—an *in vitro* model of the buccal epithelium. Pharmaceutical research 19, 169–174 (2002). 11883644 10.1023/a:1014220832384

[b55] Sander C., Nielsen H. M. & Jacobsen J. Buccal delivery of metformin: TR146 cell culture model evaluating the use of bioadhesive chitosan discs for drug permeability enhancement. Int J Pharm 458, 254–261 (2013). 24148665 10.1016/j.ijpharm.2013.10.026

[b56] Portero A., Teijeiro-Osorio D., Alonso M. J. & Remuñán-López C. Development of chitosan sponges for buccal administration of insulin. Carbohydrate Polymers 68, 617–625, doi: 10.1016/j.carbpol.2006.07.028 (2007).

[b57] Luo Y., Xu H., Huang K., Gao Z., Peng H. & Sheng X. Study on a nanoparticle system for buccal delivery of insulin. Paper presented at 2005 IEEE Engineering in Medicine and Biology Society 27th Annual International Conference, doi: 10.1109/IEMBS.2005.1615556 (2006 Jan 17). 17281326

