# Corrigendum: Pre-formulation and systematic evaluation of amino acid assisted permeability of insulin across *in vitro* buccal cell layers

**DOI:** 10.1038/srep34883

**Published:** 2016-10-14

**Authors:** Affiong Iyire, Maryam Alaayedi, Afzal R. Mohammed

Scientific Reports 6: Article number: 3249810.1038/srep32498; published online: 09 01 2016; updated: 10 24 2015

The original version of this Article contained a typographical error in the spelling of the author Maryam Alaayedi, which was incorrectly given as Maryam Alayedi. This error has now been corrected in the PDF and HTML versions of the Article.

