# Supplementary material for: Pre-formulation and systematic evaluation of amino acid assisted permeability of insulin across in vitro buccal cell layers
Source: Sci Rep. 2016 Sep 1;6:32498. doi: 10.1038/srep32498 (PMC5007592; doi:10.1038/srep32498)
Supplement: Supplementary Information [file srep32498-s1.doc]

# **Pre-formulation and systematic evaluation of amino acid assisted permeability of insulin across *in vitro* buccal cell layers**

Affiong Iyire, Maryam Alayedi, Afzal R. Mohammed*

Aston Pharmacy School, Aston University, Birmingham, B4 7ET

*a.u.r.mohammed@aston.ac.uk


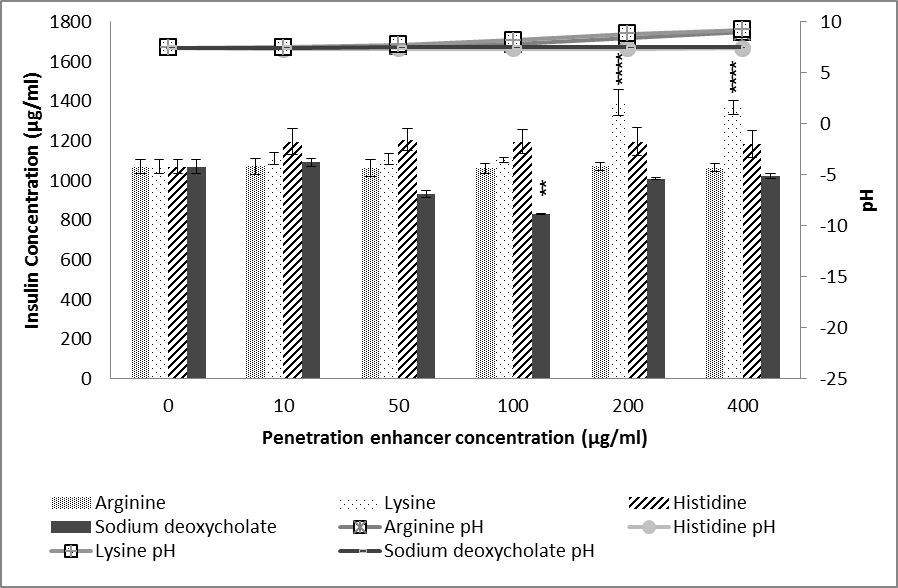


**Figure S1. Comparing the effect of basic amino acids and the bile salt, Na deoxycholate, on the solubility of insulin in HBSS at 25 ͦ C. (*) denotes level of significance using ANOVA and Turkey-Kramar post-test.**


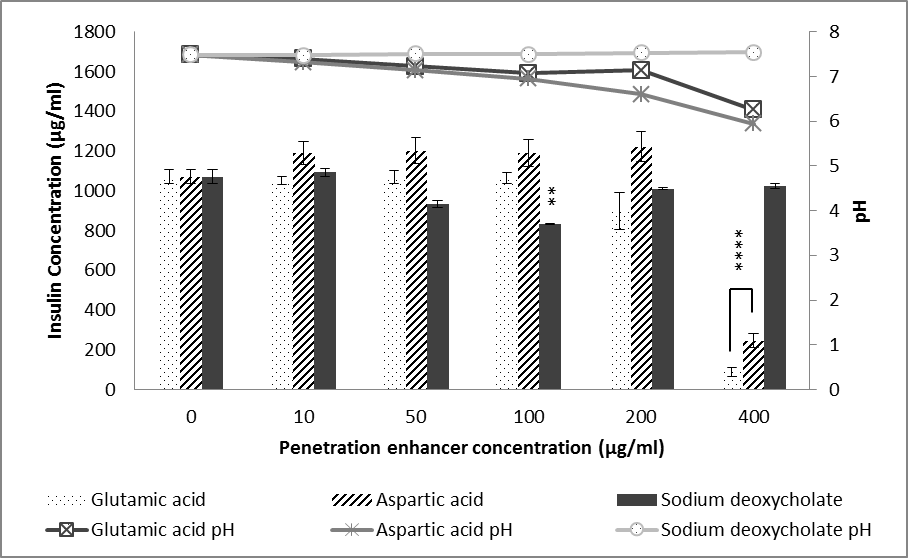


**Figure S2. Comparing the effect of acidic amino acids and Na deoxycholate on the solubility of insulin in HBSS.**


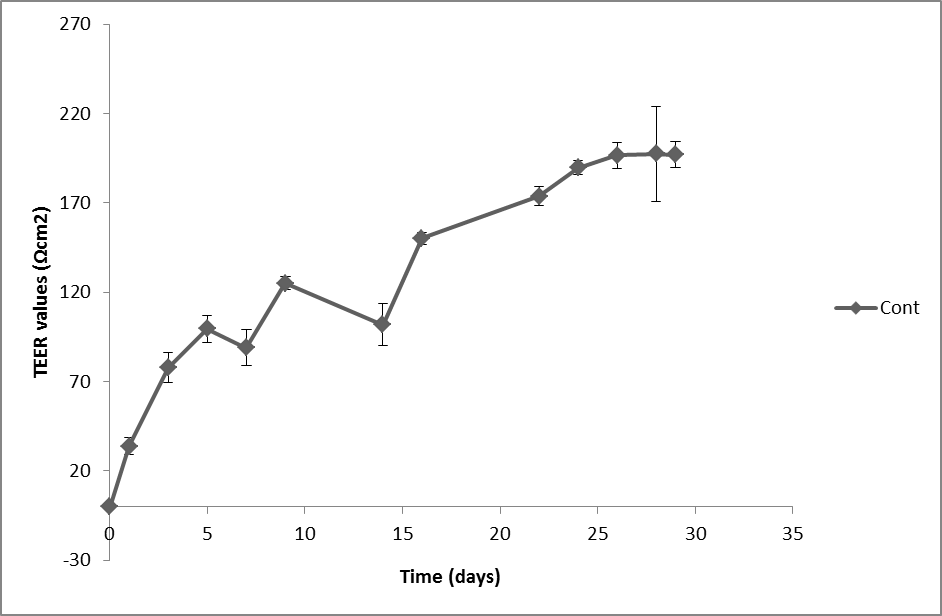


**Figure S3. Transepithelial electrical resistance results for TR146 cells grown on transwell inserts for 30 days. Cell layer integrity was found to be maintained over the time period used for the permeability studies.**


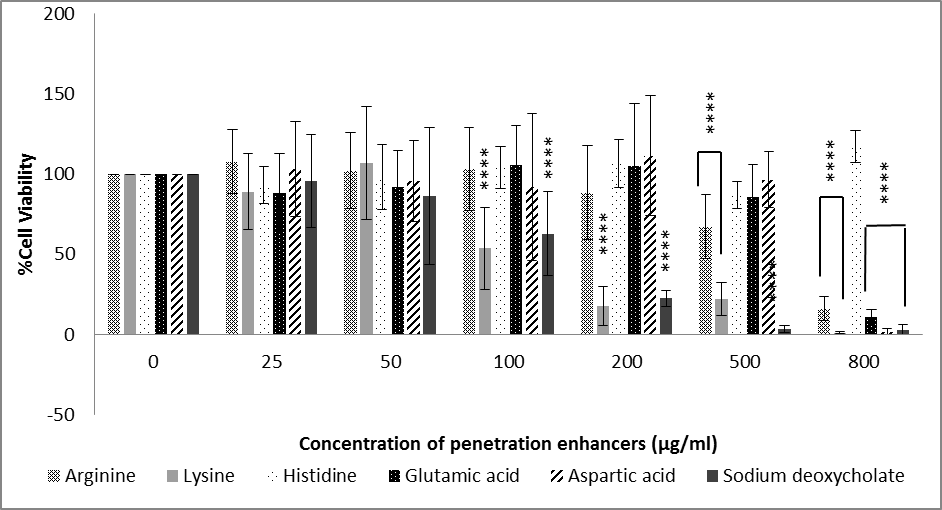


**Figure S4. Effect of penetration enhancers on the viability of TR146 cells in the presence of increasing concentrations of amino acids and Na deoxycholate, utilising the MTT assay**


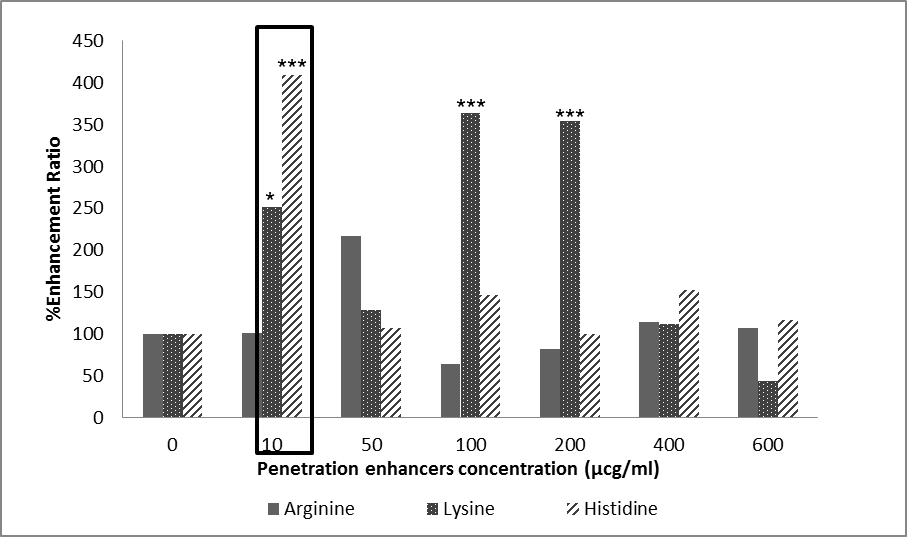


**Figure S5. Highlighting concentrations of basic amino acids that significantly enhanced insulin permeability with no toxicity to TR146 cells**


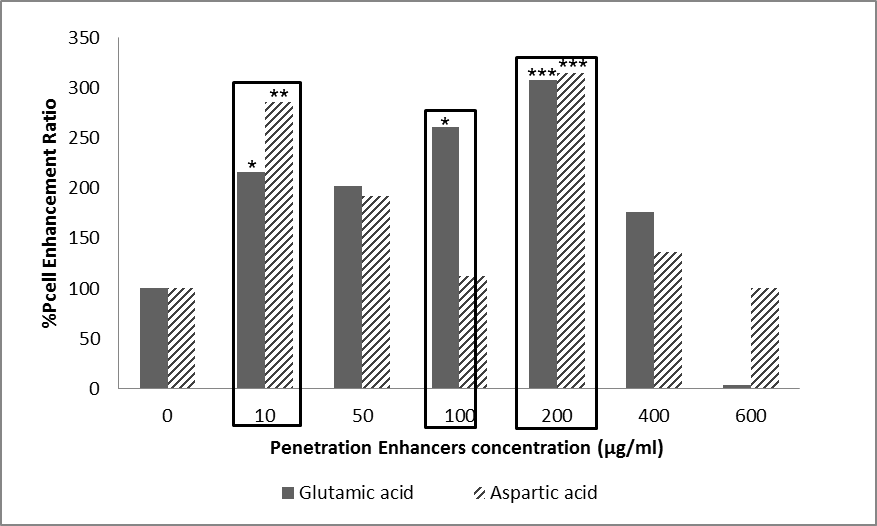


**Figure S6. Highlighting concentrations of acidic amino acids that significantly enhanced insulin permeability with no toxicity to TR146 cells**


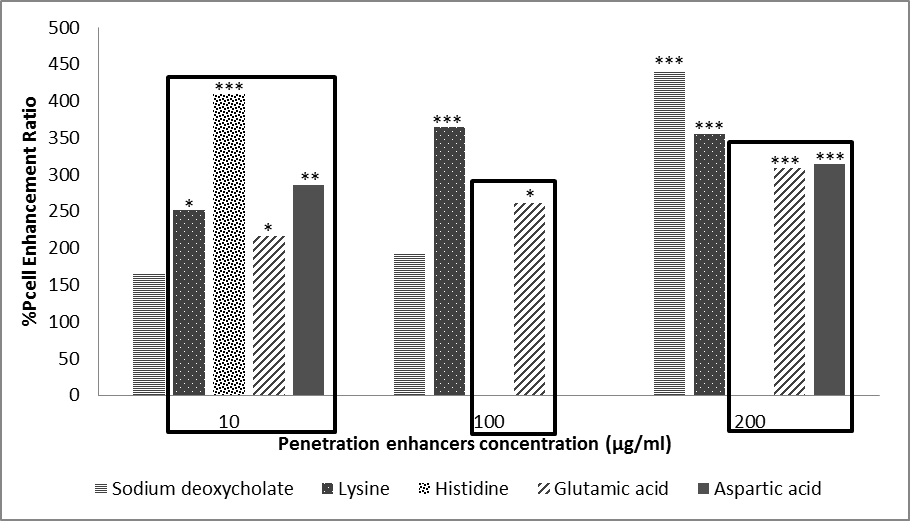


**Figure S7. Highlighting concentrations of tested penetration enhancers that significantly enhanced insulin permeability with no toxicity to TR146 cells**


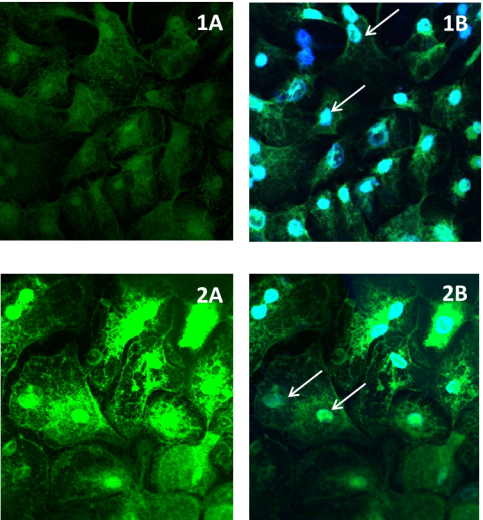


**Figure S8. Representative confocal microscopic images of FITC-insulin (A) localisation and DAPI nuclear stain (B) on TR146 cells for control (1) and in the presence of Glu 10µg/ml (2). DAPI staining confirms localisation of FITC insulin in the nucleus (white arrows), with no paracellular transport.**


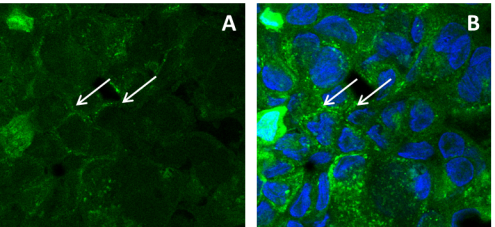


**Figure S9. Representative confocal microscopic images of FITC-dextran 20kDa localisation (A) in the intercellular spaces of TR146 cells, showing paracellular transport (white arrows). DAPI stained nuclei (blue stain) (B) confirm the absence of the paracellular marker in the nucleus.**

**Table S1. Properties of amino acids used in this study** .

| **S.N.** | **Enhancer** | **Molecular weight (g/mol)** | **Side chain** | **pKa**  **NH_3_^+^ COOH Side**  **chain** | | | **pI** |
| --- | --- | --- | --- | --- | --- | --- | --- |
| 1 | L-arginine | 174.20 | Basic | 1.82 | 8.99 | 12.48 | 10.76 |
| 2 | L-lysine | 146.19 | Basic | 2.16 | 9.06 | 10.54 | 9.74 |
| 3 | L-histidine | 155.16 | Basic/ Neutral | 1.80 | 9.33 | 6.04 | 7.59 |
| 4 | L-glutamic acid | 147.13 | Acidic | 2.10 | 9.47 | 4.07 | 3.22 |
| 5 | L-aspartic acid |  | Acidic | 1.99 | 9.90 | 3.90 | 2.77 |
| 6 | Sodium deoxycholate | 414.6 | - | - | - | - | 6.58 |

**Table S2. Effect of penetration enhancers on permeability of insulin through TR146 cell layers. P_insert_ (no cells) = 65.192 x 10^-6^ cm/s. (*) denotes level of significance (p<0.05)**

| **Formulation** | **Concentration (µg/ml)** | **P_app_ ± SD (x10^-6^)**  **(cm/sec)** | **P_cell_ ± SD (x10^-6^)**  **(cm/sec)** | **Recovery ± SD (%)** |
| --- | --- | --- | --- | --- |
| **Control** | - | 2.98 ± 0.40 | 3.12 ± 0.41 | 83.94 ± 28.87 |
| **Arg** | 10 | 3.01 ± 0.38 | 3.16 ± 0.40 | 103.03 ± 5.24 |
|  | 50 | 5.18 ± 0.40 | 5.65 ± 0.56 | 101.72 ± 2.18 |
|  | 100 | 2.11 ± 0.39 | 2.19 ± 0.42 | 97.56 ± 2.82 |
|  | 200 | 2.53 ± 1.48 | 2.64 ± 1.59 | 91.75 ± 2.70 |
|  | 400 | 3.46 ± 1.40 | 3.66 ± 1.51 | 86.63 ± 4.85 |
|  | 600 | 3.19 ± 1.75 | 3.40 ± 1.95 | 83.55 ± 3.04 |
|  |  |  |  |  |
| **Lys** | 10 | 7.08 ± 1.82* | 7.95 ± 2.12* | 81.47 ± 2.13 |
|  | 50 | 3.76 ± 1.66 | 4.02 ± 1.85 | 82.28 ± 3.03 |
|  | 100 | 9.51 ± 0.56*** | 11.19 ± 0.76*** | 83.48 ± 0.45 |
|  | 200 | 8.92 ± 4.94*** | 10.56 ± 6.59*** | 107.14 ± 7.29 |
|  | 400 | 3.31 ± 0.65 | 3.49 ± 0.71 | 89.83 ± 4.29 |
|  | 600 | 0.81 ± 0.80 | 1.25 ± 0.71 | 85.96 ± 3.22 |
|  |  |  |  |  |
| **His** | 10 | 10.7 ± 2.93*** | 12.9 ± 3.91*** | 98.76 ± 3.94 |
|  | 50 | 3.21 ± 1.19 | 3.38 ± 1.30 | 82.22 ± 19.89 |
|  | 100 | 2.89 ± 2.30 | 3.09 ± 2.48 | 90.57 ± 10.48 |
|  | 200 | 4.58 ± 2.96 | 4.99 ± 4.07 | 84.05 ± 2.33 |
|  | 400 | 3.87 ± 0.62 | 4.13 ± 0.89 | 86.16 ± 8.40 |
|  | 600 | 2.84 ± 1.12 | 2.98 ± 1.71 | 88.18 ± 4.83 |
|  |  |  |  |  |
| **Glu** | 10 | 6.12± 1.83* | 6.77E-06 ± 2.16* | 87.83 ± 2.31 |
|  | 50 | 5.83 ± 2.53 | 6.45 ± 2.99 | 85.43 ± 1.02 |
|  | 100 | 7.29 ± 2.77 | 8.27 ± 3.44* | 87.49 ± 0.52 |
|  | 200 | 9.05 ± 2.02*** | 10.61 ± 3.63** | 102.32 ± 3.63 |
|  | 400 | 5.02 ± 2.64 | 5.58 ± 3.34 | 91.28 ± 2.79 |
|  | 600 | 0.11 ± 0.01 | 0.11 ± 0.05 | 78.39 ± 1.15 |
|  |  |  |  |  |
| **Asp** | 10 | 7.71 ± 0.92** | 8.78 ± 1.19* | 95.27 ± 2.60 |
|  | 50 | 5.58 ± 2.16 | 6.13 ± 2.49 | 89.97 ± 5.76 |
|  | 100 | 3.32 ± 0.85 | 3.51 ± 0.94 | 96.92 ± 6.27 |
|  | 200 | 8.52± 2.61*** | 9.86 ± 3.32** | 85.77 ± 12.40 |
|  | 400 | 3.88 ± 0.68 | 4.14 ± 0.79 | 77.55 ± 20.39 |
|  | 600 | 2.95 ± 0.41 | 3.09 ± 0.45 | 63.59 ± 5.53 |
|  |  |  |  |  |
| **SDC** | 10 | 4.94 ± 0.54 | 5.12 ± 0.68 | 109.40 ± 12.72 |
|  | 50 | 5.72 ± 0.60 | 5.96 ± 0.87 | 104.41 ± 9.39 |
|  | 100 | 5.66 ± 0.60 | 5.90 ± 0.89 | 108.25 ± 5.63 |
|  | 200 | 11.16 ± 0.17*** | 13.55 ± 0.65*** | 89.98 ± 2.86 |
|  | 400 | 11.00 ± 0.72*** | 13.26 ± 0.59*** | 92.55 ± 2.03 |
|  | 600 | 11.13 ± 0.68*** | 13.46 ± 0.55*** | 95.41 ± 1.35 |

**Table S3. Transepithelial electrical resistance (TEER) measurements for enhancers before and after permeability experiments through TR146 cell layers**

| **Formulation** | **Concentration (µg/ml)** | **TEER measurements before ± SD (Ω/cm^2^)** | **TEER measurements after ± SD (Ω/cm^2^)** | **Recovery**  **(%)** |
| --- | --- | --- | --- | --- |
| **Control** | - | 254.99 ± 75.45 | 254.12 ± 83.06 | 99.66 |
| **Arg** | 10 | 255.73 ± 60.48 | 266.56 ± 83.77 | 104.23 |
|  | 50 | 223.25 ± 25.88 | 281.49 ± 60.83 | 126.09 |
|  | 100 | 215.16 ± 46.03 | 238.68 ± 48.21 | 110.93 |
|  | 200 | 265.19 ± 98.54 | 275.40 ± 53.04 | 103.85 |
|  | 400 | 255.11 ± 72.29 | 281.74 ± 80.82 | 110.44 |
|  | 600 | 324.43 ± 112.63 | 260.09 ± 90.12 | 80.17 |
|  |  |  |  |  |
| **Lys** | 10 | 275.27 ± 58.57 | 299.91 ± 97.82 | 108.95 |
|  | 50 | 317.21 ± 54.83 | 294.44 ± 53.03 | 107.18 |
|  | 100 | 447.00 ± 69.32 | 274.4 ± 41.58**** | 61.39 |
|  | 200 | 337.37 ± 86.83 | 299.91 ± 54.94 | 88.90 |
|  | 400 | 296.55 ± 70.94 | 283.86 ± 65.75 | 95.72 |
|  | 600 | 279.63 ± 38.03 | 304.27 ± 69.22 | 108.81 |
|  |  |  |  |  |
| **His** | 10 | 284.60 ± 69.65 | 338.99 ± 71.12 | 119.11 |
|  | 50 | 283.61 ± 102.74 | 271.04 ± 70.50 | 95.57 |
|  | 100 | 366.86 ± 85.50 | 298.54 ± 62.06 | 81.38 |
|  | 200 | 394.24 ± 69.75 | 283.86 ± 48.88** | 72.00 |
|  | 400 | 348.82 ± 52.10 | 273.53 ± 78.09 | 78.42 |
|  | 600 | 308.25 ± 50.88 | 362.76 ± 58.50 | 117.68 |
|  |  |  |  |  |
| **Glu** | 10 | 370.60 ± 97.80 | 336 ± 147.11 | 90.66 |
|  | 50 | 381.42 ± 96.48 | 320.57 ± 59.24 | 84.05 |
|  | 100 | 459.2 ± 102.68 | 286.72 ± 43.37 | 62.44 |
|  | 200 | 343.96 ± 86.82 | 252.37 ± 52.52 | 73.37 |
|  | 400 | 350.93 ± 102.59 | 283.11 ± 38.77 | 80.67 |
|  | 600 | 303.77 ± 87.62 | 337 ± 72.72 | 110.94 |
|  |  |  |  |  |
| **Asp** | 10 | 295.06 ± 25.06 | 295.56 ± 98.81 | 100.17 |
|  | 50 | 275.27 ± 27.47 | 292.44 ± 64.82 | 106.24 |
|  | 100 | 289.71 ± 80.61 | 258.84 ± 46.01 | 89.35 |
|  | 200 | 349.44 ± 104.69 | 243.66 ± 50.88 | 69.73 |
|  | 400 | 379.80 ± 114.17 | 251.25 ± 85.45 | 66.15 |
|  | 600 | 558.51 ± 67.52 | 283.61 ± 108.87*** | 50.78 |
|  |  |  |  |  |
| **SDC** | 10 | 211.33 ± 38.58 | 222.22 ± 40.91 | 105.15 |
|  | 50 | 234.56 ± 48.87 | 195 ± 37.08 | 83.14 |
|  | 100 | 179.67 ± 48.87 | 199.89 ± 44.80 | 111.26 |
|  | 200 | 252.78 ± 45.86 | 188.89 ± 39.91* | 74.73 |
|  | 400 | 244.56 ± 87.71 | 172 ± 61.47* | 70.33 |
|  | 600 | 224.22 ± 25.82 | 155.33 ± 57.05** | 67.05 |
